# Genotoxicity and Micronucleus Formation as a Result of Panoramic Radiography in Epithelial Cells of the Buccal Mucosa: A Cross‐sectional Study in Adults

**DOI:** 10.1002/cre2.915

**Published:** 2024-08-07

**Authors:** Nakisa Torabinia, Mojdeh Mehdizadeh, Forooz Keshani, Mohammadreza Mehdizadeh, Parisa Soltani, Gianrico Spagnuolo

**Affiliations:** ^1^ Department of Oral and Maxillofacial Pathology, Dental Research Center, Dental Research Institute, School of Dentistry Isfahan University of Medical Sciences Isfahan Iran; ^2^ Department of Oral and Maxillofacial Radiology, Dental Implants Research Center, Dental Research Institute, School of Dentistry Isfahan University of Medical Sciences Isfahan Iran; ^3^ Department of Oral and Maxillofacial Pathology, Dental Materials Research Center, Dental Research Institute, School of Dentistry Isfahan University of Medical Sciences Isfahan Iran; ^4^ Students Research Committee, School of Dentistry Isfahan University of Medical Sciences Isfahan Iran; ^5^ Department of Neurosciences, Reproductive and Odontostomatological Sciences University of Naples “Federico II” Naples Italy

**Keywords:** cytology, genotoxicity, micronucleus test, panoramic radiography, radiation

## Abstract

**Objectives:**

To determine the genetic effects of panoramic radiography on the epithelial cells of the buccal mucosa by examining the micronucleus formation in these cells.

**Materials and Methods:**

In this cross‐sectional study, exfoliative cytology samples were prepared from the buccal mucosa of 36 patients immediately before and 10 days after panoramic radiography. The samples were prepared using liquid‐based cytology with Papanicolaou staining. The slides were simultaneously evaluated by two expert pathologists and the ratio of the number of cells with micronuclei to the total number of cells on the slide was reported as a percentage. Data analysis was done using paired‐samples *T* test, Pearson's correlation coefficient, and covariance analysis (*α* = 0.05).

**Results:**

The study sample consisted of 24 (66.67%) males and 12 females (33.33%) with a mean (SD) age of 27.36 (8.19) years. The frequency of cells with micronucleus before and after panoramic radiography was not statistically different (*p* = 0.468). Additionally, the frequency of micronucleated cells was not correlated with age (*p* = 0.737) and sex (*p* = 0.211).

**Conclusion:**

Panoramic exposure slightly increased the frequency of cells with micronucleus in epithelial cells of the buccal mucosa. However, this increase was not statistically significant.

## 1 Introduction

Panoramic radiography is one of the most frequently obtained radiographs in dentistry, providing a gross overview of the dentition and surrounding structures. It is an easily accessible and low‐cost radiographic modality with a relatively low radiation dose (Izzetti et al. 2021). However, as with all X‐ray‐based modalities, the risk of biological damages should not be overlooked.

The current paradigm in radiation safety is based on the linear no‐threshold (LNT) model for dose–response (Doss 2013). The LNT model argues that the risk of cancer in radiation doses less than 100 mSv is proportional to the received dose and that there is no threshold below which there is no risk of added cancer. Although not a scientifically proven fact, the LNT model provides a useful basis for radiation protection organizations to regulate the dose limits of individuals exposed to low‐dose ionizing radiation (Weber and Zanzonico 2017).

During panoramic exposure, the oral mucosal epithelium is inevitably exposed to radiation (Santos et al. 2022). The nucleus of the cells is much more sensitive to radiation‐induced damage compared to the cytoplasmic structures (Santhosh et al. 2020). Genetic damage, such as chromosome aberration and micronucleus formation, are among the mechanisms involved in carcinogenesis (Baumgart et al. 2016). Micronuclei are segments of deoxyribonucleic acid (DNA) aberrated as a result of damage to chromosomes or chromatids. These small DNA segments are separated from the main nucleus during mitosis and form a small rudimentary nucleus in the cytoplasm. In recent years, micronucleus formation has been considered a biomarker for the evaluation of chromosomal damage, genome instabilities, and the risk for malignancies (Sandhu, Mohan, and Kumar 2015; Buajeeb et al. 2007; Li et al. 2018). Evaluation of micronucleus formation in exfoliated cells is a reliable marker for genetic toxicity and early detection of premalignant and malignant lesions (Jadhav, Gupta, and Ahmed Mujib 2011; Aggarwal et al. 2019).

Several studies have been performed to address the cytotoxic effects of X‐ray radiation on oral mucosal cells. Some studies reported a significant increase in the number of micronucleated cells after panoramic exposure (Santos et al. 2022; Li et al. 2018; Preethi, Chikkanarasaiah, and Bethur 2016), while others found no significant difference in pre‐ and postexposure samples (Santhosh et al. 2020; Aggarwal et al. 2019; De Souza et al. 2021). Recent advances in cytology enable preparations of smears using liquid‐based cytology, offering a higher sensitivity compared to conventional cytology (Honarvar et al. 2022). However, this technique has not been used in older studies investigating the genotoxic effects of panoramic exposure on oral epithelial cells. Therefore, the present study aimed to evaluate micronucleus formation in oral mucosal epithelial cells as a result of panoramic radiography using liquid‐based cytology. The research hypothesis was that exposure to panoramic radiography does not lead to a significant change in the amount of micronucleated cells in the buccal mucosa.

## 2 Methods

This cross‐sectional study was approved by the Research Ethics Committee at Isfahan University of Medical Sciences (#IR.MUI.RESEARCH.REC.1401.297, approval date: 14/1/2023). The objectives of the study were explained for the individuals and informed consent was obtained from all the participants. The principles of the Declaration of Helsinki were followed.

### 2.1 Patient Selection

According to the following formula, with a minimum sample of 36 individuals, there is an 80% possibility (1 −* β* = 0.80) that a difference of 1.1% in the number of micronucleated cells between the pre–post mean values is significant at a level of 0.05 (α = 0.05).

$$n=\frac{2\times {\left(Z_{1-\frac{\alpha }{2}}+Z_{1-\beta }\right)}^{2}(1-\rho )\sigma^{2}}{d^{2}}.$$

The study was performed on patients attending the Department of Oral and Maxillofacial Radiology at Isfahan School of Dentistry for obtaining panoramic radiography for dental reasons unrelated to this study from February to May 2023. Inclusion criteria were: (1) individuals aged between 18 and 55, (2) nonsmokers, (3) those without a history of drug abuse, (4) those without removable dentures, (5) those without oral ulcers or lesions, and (6) those without the history of using mouthwash, alcohol, and antibiotics in the last 10 days, and (7) those who did not take radiographs in the last 6 months. Exclusion criteria were: (1) individuals who did not come back in 10 days for the second biopsy, (2) those willing to exit the study for any reason, (3) those who have been exposed for other radiographic images during the 10‐day period, (4) those who have smoked during this 10‐day period, and (5) those with consumption of antibiotics, alcohol, and mouthwash during the 10‐day period. Inclusion and exclusion criteria were enforced to control other confounding agents that are suggested to cause genotoxic effects before the study and during the 10‐day period, as much as possible (Milić et al. 2019; Papis, Davies, and Jha 2011; Rocco, Peluso, and Stingo 2012; Akhtar et al. 2022).

### 2.2 Exfoliative Cytology

**Figure 1:**
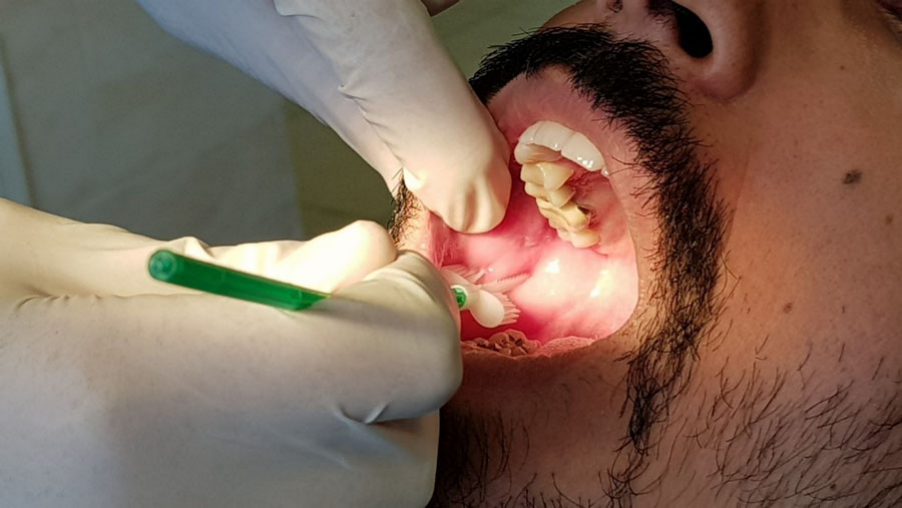
Exfoliative cytology of the right buccal mucosa using a cytobrush.

Exfoliative biopsy was performed twice in this study: once immediately before and once 10‐days after obtaining the panoramic radiograph. For each round of biopsy, the patients were asked to rinse their mouth with water. Then, a sterile cytobrush (Osture Tajhiz Darman, Isfahan, Iran) was used with slight pressure and a sweeping motion on the right buccal mucosa (Figure 1). Thereafter, the head of the cytobrush was removed and placed in a fixative vial containing 10 mL of ethanol‐based preservative solution. The containers were coded and sent for cytological analysis.

### 2.3 Obtaining the Panoramic Radiograph

The panoramic radiograph was obtained using Promax (Planmeca, Helsinki, Finland) device with the exposure parameters of 66 kVp, 7.1 mA, and 15.8 s. Patients were positioned in the device with their Frankfurt plane horizontal to the floor and their midsagittal plane perpendicular to the floor.

### 2.4 Preparation of the Slide

The fixative vial containing the preservative solution and the cytobrush with the scraped cells were shaken to release the cells from the cytobrush into the solution. Thereafter, the solution (10 mL) was transferred to the filtration vial and placed in the liquid‐based cytology system (E‐prep, PATHtech, Jeonju‐si, South Korea). The filter had a 2 cm diameter and thus, the cells were distributed in a circle with a diameter of 2 cm on the slide. Positive charged slide with poly‐l‐lysine coating was used in this study. The slide was then extracted from the system and dried at room temperature. Thereafter, it was placed for 10 min in 100% methanol for fixation.

Followed by that, the samples were stained using Papanicolaou staining. The slides were first in contact with hematoxylin for 10 min to allow staining of the nuclei. Then, the slide was washed with water and dipped in a solution containing 240 mL methanol, 60 mL sterile water, and 2 mL hydrochloric acid 80%. The slide was washed again with water. This process allowed for the washout of hematoxylin dye from cytoplasm and higher visibility of the cells. Thereafter, the slide was immersed in the following solutions in order: methanol 100%, Orange‐Green‐6 dye for 4 min, methanol 85%, methanol 75%, Eosin‐Azure‐50 dye for 4 min, methanol 85%, methanol 75%, and ethanol 100%. Thereafter, the slides were dried at room temperature and mounted using xylene (MilliporeSigma, Burlington, MA, USA), Entellan (MilliporeSigma, Burlington, MA, USA) and cover glass.

### 2.5 Evaluation of the Slides

The slides were viewed by two oral and maxillofacial pathologists (N.T. and F.K. with 20 and 10 years of experience, respectively) simultaneously using a light microscope (Olympus CH4, Olympus Life Sciences, Tokyo, Japan) with ×400 magnification. The observers did not know if the slide was a before or after exposure specimen. Identification of micronucleus was based on the criteria described by Sarto et al. (1987) The number of the cells with micronucleus were divided by the number of the entire cells on the slide in percentage.

### 2.6 Statistical Analysis

The data were presented using mean and standard deviation (SD). Analysis of the data was performed using paired‐samples *T* test and Pearson correlation coefficient. Covariance analysis was used to investigate the correlation with age and sex. SPSS (version 22, IBM Statistics, Armonk, NY, USA) was used for statistical analysis. The significance level was set at 0.05.

## 3 Results

**Figure 2:**
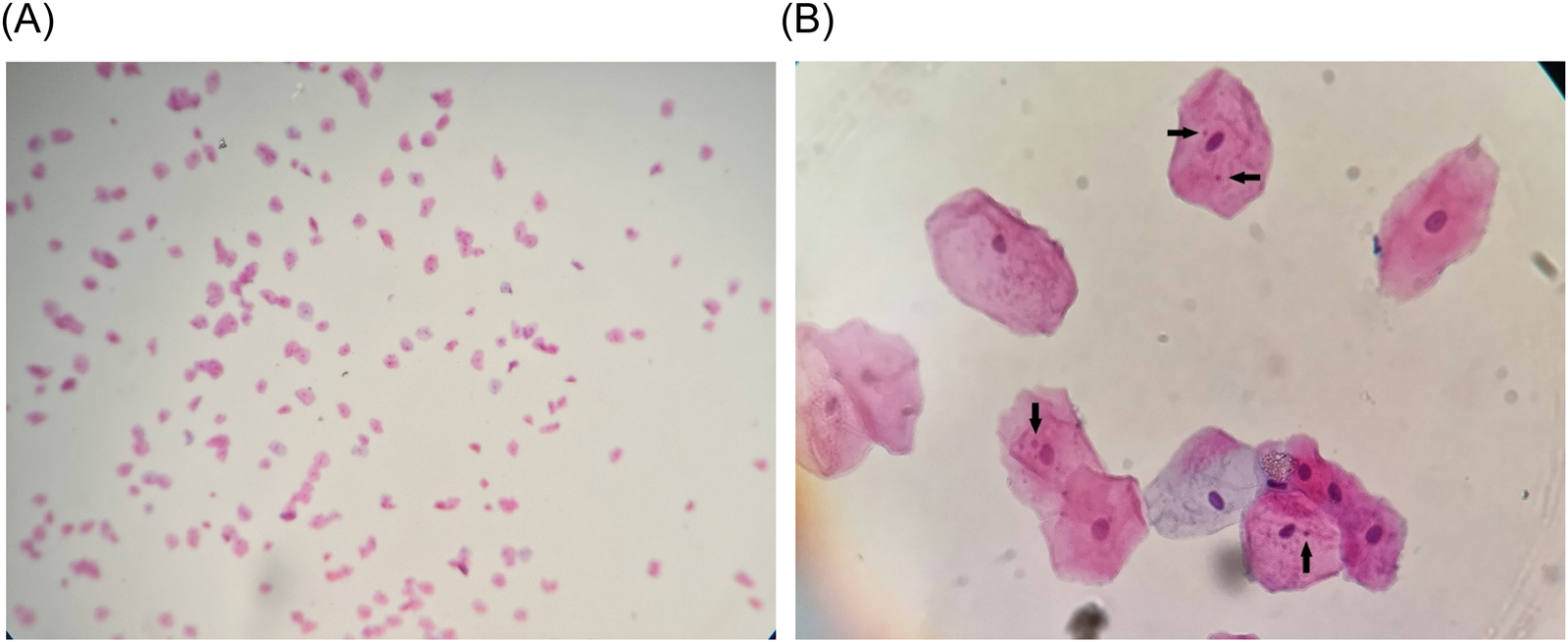
(A) Cytological sample of exfoliated buccal epithelial cells (×10) and (B) presence of micronucleus (arrows) in the exfoliated cells (×400).

Thirty‐six individuals initially participated in the study and all of them returned later after 10 days for the second biopsy. The study sample was comprised of 24 (66.67%) males and 12 females (33.33%) with a mean (SD) age of 27.36 (8.19). Figure 2 shows a slide containing cells with micronucleus.

**Figure 3:**
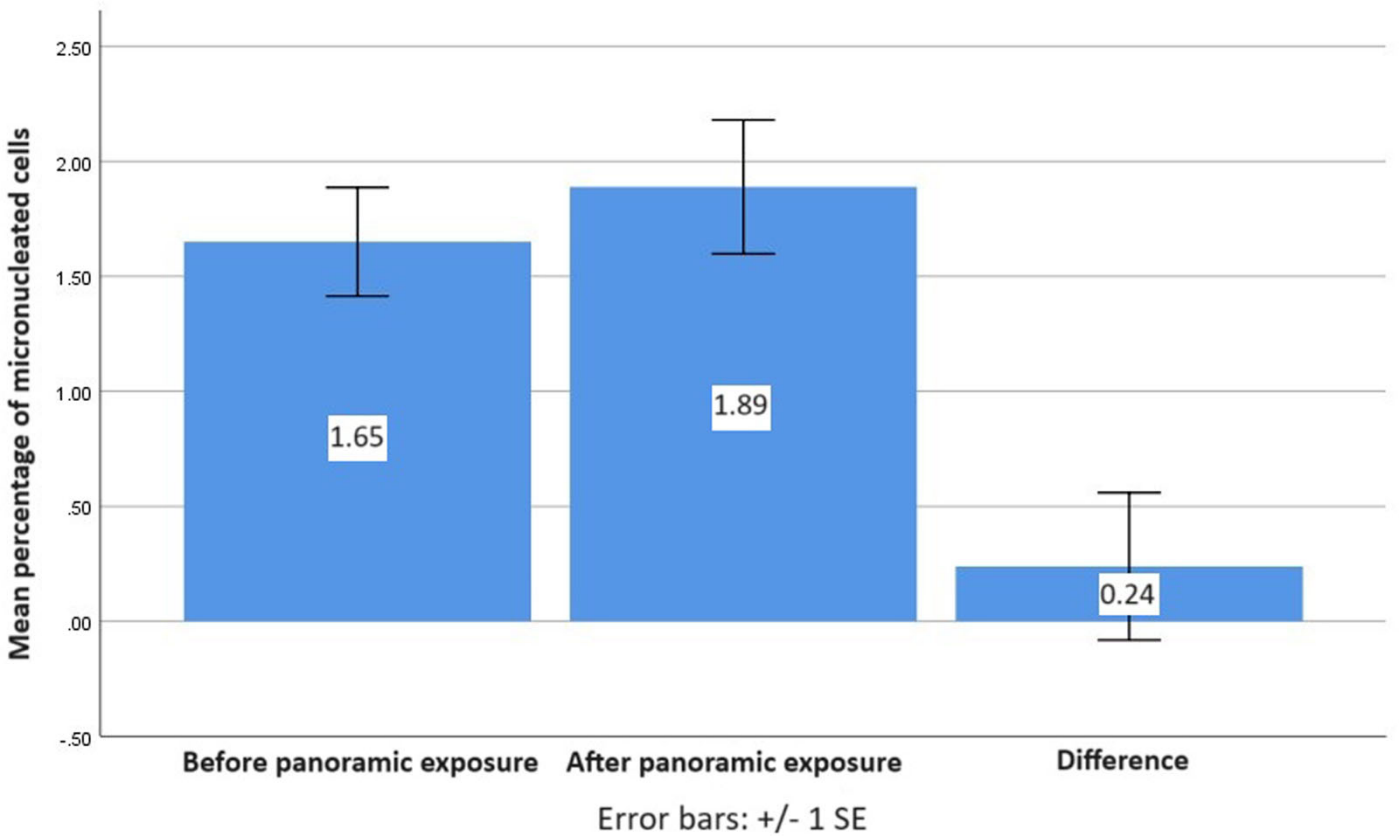
Diagram depicting mean percentage of micronucleated cells before and 10 days after panoramic exposure and the difference between these values.

The mean percentage of micronucleus before and after panoramic exposure was 1.65 and 1.88, respectively. Despite the slight increase (0.24%) in the ratio of cells with micronucleus after panoramic exposure, the paired‐samples *T*‐test showed no significant difference existed between the percentages of cells with micronucleus before (1.65%) and after (1.89%) exposure (*p* = 0.468, Table 1 and Figure 3). Additionally, Pearson's correlation coefficient showed that age is not significantly correlated to micronucleus frequency before (*r* = 0.012, *p* = 0.946) and after (*r* = 0.065, *p* = 0.707) exposure. To confirm this, covariance analysis revealed that the effect of age (*p* = 0.737) and sex (*p* = 0.211) is not statistically significant.

**Table 1:** Mean and standard deviation (SD) values of micronucleus frequency.

	Mean (SD)	*p* value
Female		
Before	1.79 (1.57)	0.361
After	2.56 (2.59)	
Male		
Before	1.57 (1.36)	0.937
After	1.60 (1.07)	
Total		
Before	1.65 (1.41)	0.468
After	1.88 (1.74)	

## 4 Discussion

The results of the study showed that the percentage of oral epithelial cells with micronucleus before and after panoramic radiography is not statistically different. Additionally, the percentage of micronucleus formation was not correlated with age and sex.

In 2015, Aggarwal et al. (2019) evaluated the genetic effects of panoramic radiography on buccal epithelial cells in 20 pediatric patients. The cytology samples were collected before and 10 days after panoramic exposure and stained using rapid Papanicolaou staining. The results revealed that mean micronucleus formation was not significantly different between the two samples. This finding of Agarwal et al. in pediatric patients is consistent with the findings of our study on adult individuals.

In another study in 2020, Santhosh et al. (2020) studied the smears taken from the buccal mucosa of adult participants using modified Feulgen‐Rossenbeck staining. Similar to the present study, they observed no significant increase in the number of micronuclei cells present before and after panoramic radiography. Additionally, they reported than no significant difference existed between males and females in the frequency of micronuclei. However, in contrast with this study, they found a positive correlation between age and the frequency of micronuclei. The settings in the study by Santhosh et al. was different from this study. They included 30 individuals without exposure to panoramic imaging and another 30 participants, different from the original group, who were exposed to panoramic imaging 12 days ago. Additionally, the age range of the individuals is not clear in the study of Santhosh et al. In the inclusion criteria they mention an age range of 24–65 years, while in the results it is noted that the majority of patients aged between 20 and 25 years. The difference between the findings of the two studies regarding correlation with age can be attributed to these factors.

Anbumeena et al. (2021) in 2021, investigated the genotoxicity and cytotoxicity of panoramic imaging in five age groups, each containing 12 individuals. Buccal mucosal smear was obtained from participants once before and once 7–10 days after panoramic radiography and stained using Papanicolaou and periodic acid‐Schiff staining. The results indicated that although panoramic imaging slightly increased the number of micronuclei, the difference was not statistically significant before and after exposure. This finding is in line with that of the present study. Additionally, similar to the present study, Anbumeena et al. reported that age is not correlated with formation of micronuclei in epithelial cells of the buccal mucosa. The correlation of sex with mean micronucleus frequency was not investigated in the study of Anbumeena et al.

Despite the observations in this study, some studies reported a significant increase in micronucleus formation after panoramic imaging. In 2014, Arora, Devi, and Wazir (2014) evaluated the buccal mucosal and gingival cells of 53 individuals before and 10 days after panoramic radiography. They prepared the smears using Giemsa staining. They reported a significant increase in the frequency of buccal mucosal cells with micronucleus after panoramic exposure, while this increase was not statistically significant for gingival cells. They have used higher exposure parameters compared to the present study, which can justify the different observations of the two studies. Similar to the present study, Arora et al. found no significant correlation between sex and micronucleus formation. Moreover, no association between the frequency of micronucleus formation and age was found in the buccal mucosal cells.

In another study, Sandhu, Mohan, and Kumar (2015) in 2015 compared the genotoxic effects of conventional and digital panoramic radiography in 100 females. They used Giemsa solution for staining a smear of buccal mucosal cells immediately before and 10 days after panoramic exposure. They observed that both conventional and digital panoramic imaging leads to a significant increase in the mean frequency of micronuclei. However, this increase in conventional radiography was much more than in digital imaging. Other studies including Preethi, Chikkanarasaiah, and Bethur (2016), Li et al. (2018), and Kaur et al. (2020) also reported a significant increase in micronuclei after panoramic exposure. The differences between the findings might be due to different age range and different exposure settings. Additionally, the number of participants in the studies by Preethi et al. and Li et al. were less than the present study.

In 2021, De Souza et al. (2021) performed a systematic review and meta‐analysis to find out if panoramic exposure induces cytogenetic damage to oral cells. This study failed to demonstrate the association between the frequency of micronucleus formation and exposure to panoramic radiography. In 2022, Santos et al. (2022) performed a systematic review and meta‐analysis on the topic. They concluded that panoramic radiographs can cause genotoxic damage in the oral epithelium but with a small effect size. However, the quality of the investigated evidence was considered low. The results of another systematic review and meta‐analysis by Malacarne et al. (2023) in 2023 showed that indicated that radiographic examinations in children cause genotoxic effects with a very low degree of evidence and high heterogeneity. However, this study involved different types of radiographic modalities including cone beam computed tomography, which has a higher radiation dose compared to panoramic exposure (Mehdizadeh et al. 2015). It is worth mentioning that in addition to different radiation doses and different demographics of participants, variations in methods for biopsy, as well as fixing, staining, and counting of the cells can lead to different results in the studies.

Epithelial cells of the buccal mucosa are exposed to radiation during dental radiographic examinations. These cells are highly proliferative and thus are sensitive to radiation‐induced genetic damage. Evaluation of the frequency of micronucleated cells is a sensitive method for investigation of genetic damage to these cells (Malacarne et al. 2023). The exfoliative nature of epithelial cells plays a fundamental role in the biopsy technique. Chromosomal aberrations resulting in micronucleus formation usually occur in the proliferating basal cells of the epithelium. Due to their high turnover, these cells need 7–21 days to reach the superficial layers of the epithelium and be collected during exfoliative cytology. Therefore, 1–3 weeks after the genotoxic event, it is expected to observe the micronuclei in the exfoliated cells of the oral mucosa (Waingade and Medikeri 2012).

In this study, instead of preparation of a direct cellular smear, liquid‐based cytology was used for epithelial cells of the buccal mucosa and the slides were prepared using the filtration and precipitation method. In contrast to the direct smear used in older studies, this technique allows for formation of a single‐layer uniform smear of cells without mucosa and blood remnants. Therefore, the cells will be visualized more clearly and the sensitivity of the test will be increased (Honarvar et al. 2022; Hashmi et al. 2020). Conventional cytology techniques, despite diligent preparation, result in the transfer of only approximately 20% of cells to the slide, often obscured by other materials. In contrast, liquid‐based cytology offers several advantages. Liquid‐based cytology provides a clearer background, minimizing cellular overlap and allows for more efficient and timely analysis compared to conventional smears (Dasgupta 2023). These benefits make the liquid‐based technique a preferred choice for screening and detection of premalignant or malignant changes and conditions using cytology. In the present study, inclusion and exclusion criteria were selected to control the confounding factors that are suggested to cause genotoxicity when exposed to cells, including antibiotics, mouthwashes, alcohol, cigarette smoke, and additional radiation (Milić et al. 2019; Papis, Davies, and Jha 2011; Rocco, Peluso, and Stingo 2012; Akhtar et al. 2022).

A limitation of this study was the lack of measurement of the effective doses of the buccal mucosa. However, the exposure parameters and patient positioning were standardized. Additionally, in this study, samples were collected only from the buccal mucosa. Investigation of the effects of panoramic exposure on blood lymphocytes could also add more insights to the radiation‐induced genotoxicity in individuals exposed to dental diagnostic radiographs. In addition, possible genotoxic effects of exposure to other radiographic modalities can also be investigated in future research studies. As mentioned earlier, the evidence on whether or not panoramic exposure is genotoxic for oral mucosal cells is divided. The authors believe that the studies reporting the genotoxic effects of panoramic images should not be overlooked. According to the LNT model of dose–response, no threshold exists below which there is no risk for added cancer. Therefore, the principle of “as low as reasonably achievable (ALARA)” should always be considered when subjecting individuals to ionizing radiation for diagnostic purposes. A detailed and comprehensive clinical examination should always precede exposing dental patients to ionizing radiation.

## 5 Conclusion

Panoramic exposure slightly increased the frequency of cells with micronucleus in epithelial cells of the buccal mucosa. However, this increase was not statistically significant.

## Author Contributions

N.T. supervised the study, performed the cytological procedures, and wrote the initial draft; M.M. designed the study, obtained the panoramic radiographs, and critically revised the original draft; F.K. performed the cytological procedures, and wrote the initial draft; M.M. helped in designing the study, performed the exfoliative biopsy, and wrote the initial draft; P.S. interpreted the data, and wrote the initial draft; G.S. interpreted the data, and critically revised the original draft; All authors approved the final manuscript.

## Ethics Statement

This cross‐sectional study was approved by the Research Ethics Committee at Isfahan University of Medical Sciences (#IR.MUI.RESEARCH.REC.1401.297, approval date: 14/1/2023). The objectives of the study were explained for the individuals and informed consent was obtained from all the participants. The principles of the Declaration of Helsinki were followed.

## Conflicts of Interest

The authors declare no conflicts of interest.

## Data Availability

The data supporting this manuscript is available from the corresponding author upon reasonable request.
